# Efficacy of enteral ticagrelor in hypothermic patients after out-of-hospital cardiac arrest

**DOI:** 10.1007/s00392-015-0925-1

**Published:** 2015-10-27

**Authors:** Lisa M. Tilemann, Jan Stiepak, Thomas Zelniker, Emanuel Chorianopoulos, Evangelos Giannitsis, Hugo A. Katus, Oliver J. Müller, Michael Preusch

**Affiliations:** Department of Internal Medicine III, University Hospital Heidelberg, Im Neuenheimer Feld 410, 69120 Heidelberg, Germany; DZHK (German Centre for Cardiovascular Research), Partner Site, Heidelberg/Mannheim, Germany

**Keywords:** Neuroprotective hypothermia, Out-of-hospital cardiac arrest, Ticagrelor, P2Y12 antagonists, Myocardial infarction

## Abstract

**Introduction:**

Delivery of crushed ticagrelor via a nasogastric tube is a widely spread off-label use in unconscious patients following out-of-hospital cardiac arrest (OHCA). Notwithstanding the importance of a potent dual antiplatelet therapy in these patients, the efficacy of crushed ticagrelor after OHCA has not been established yet.

**Methods:**

In a prospective, single-center, observational trial, 38 consecutive MI patients after OHCA were included. 27 patients (71.1 %) underwent mild induced hypothermia. The primary outcome was platelet inhibition at 24h measured by impedance aggregometry.

**Results:**

There was sufficient platelet inhibition in most patients after OHCA. In all hypothermic patients, there was an adequate platelet inhibition by ticagrelor at 24 h (*p* < 0.001). 15 patients (39.5 %) had significant gastroesophageal reflux and one patient with significant reflux had inadequate platelet inhibition at 24 h. There were no stent thrombosis or recurrent atherothrombotic events in these patients.

**Conclusion:**

Administration of crushed ticagrelor via a nasogastric tube reliably inhibited platelet function in vitro and in vivo regardless of the presence of hypothermia in MI patients. Thus, platelet inhibition can be reliably achieved in MI patients during neuroprotective hypothermia following OHCA.

## Introduction

Cardiac diseases such as acute myocardial infarction (MI) are the leading cause for out-of-hospital cardiac arrest (OHCA) [1]. An effective dual antiplatelet therapy is crucial for the successful clinical management of these high-risk patients. In the PLATelet inhibition and patient outcomes (PLATO) trial, ticagrelor was associated with significant reductions in cardiovascular events, cardiovascular mortality, and all-cause mortality in patients presenting with acute coronary syndromes (ACS) compared to clopidogrel [2].

The post-cardiac arrest phase is a complex combination of various processes, including myocardial dysfunction, systemic ischemia/reperfusion response and brain injury [3–5]. These effects lead to an activation of endogenous coagulation and anticoagulation, acidosis, and pro-inflammatory mechanisms, which can alter platelet count and function [6–9]. Systemic hypoxemia also involves the GI tract, potentially reducing the absorption and metabolization of enterally applied drugs. Malabsorption may be even worsened by the application of sedatives and analgesics such as opioids. In addition, treatment strategies such as therapeutic hypothermia can affect platelet function as well as the response to medication, especially if enzymatic activation of the drug is required [6, 10, 11]. Acidosis and pro-inflammatory processes in the post-cardiac arrest phase can have an impact on blood coagulation and platelet function [4, 12, 13]. In addition, activation of the coagulation and anticoagulation system as well as simultaneous activation of endogenous fibrinolysis and anti-fibrinolysis may contribute to microcirculatory reperfusion disorders [14, 15]. During the post-cardiac arrest phase with ongoing hypothermia, platelet inhibition by clopidogrel is almost nonexistent [16–18]. Unlike clopidogrel, ticagrelor does not require cytochrome p450 (CYP) conversion and there are no genetic polymorphisms known that may result in a loss of function, and therefore be of disadvantage in patients after OHCA [19].

Cardiac arrest survivors caused by a MI are particularly in need for a potent antithrombotic medication, and ticagrelor is commonly used in these patients. However, application, metabolism, and action conditions of ticagrelor during the early post-cardiac arrest phase differ substantially from the setting in previous studies that tested ticagrelor in cardio-circulatory stable MI patients. Therefore, the aim of this study was to evaluate the efficacy of ticagrelor in MI patients with and without therapeutic hypothermia following OHCA.

## Methods

The study was approved by the Ethics Committee of Heidelberg (approval reference number S-388/2011) and complies with the principles laid down in the Declaration of Helsinki. Informed consent was obtained from the legal guardians or the next kin. Surviving patients, who regained a good mental state, confirmed the consent to participate in the study later on.

### Design

We conducted a prospective, single-center, observational study. Patients after OHCA and suspected ACS were admitted to the intensive care unit of the Department of Cardiology of the University Hospital of Heidelberg and added to the database of the Heidelberg Resuscitation Registry. Prior to admission, patients were treated with unfractioned intravenous heparin and intravenous ASA. If the suspected diagnosis was ACS (e.g., by anamnesis or changes in the electrocardiogram), patients were loaded with ticagrelor. The loading dose was 180 mg followed by 90 mg twice a day. Ticagrelor tablets were crushed, suspended in 10–20 mL aqua, and administered with a standard 20 mL polyethylene/polypropylene oral dispenser syringe (Exadoral^®^, B. Braun Melsungen AG, Melsungen, Germany) via a standard 15 French polyurethane nasogastric tube (Freka^®^ Tube, Fresenius Kabi AG, Bad Homburg, Germany) followed by 10 mL of flush with aqua. If possible ticagrelor was administered directly after admission. Patients underwent a cardiac catheterization and intensive care treatment according to the standards of the intensive care unit. Patients underwent mild therapeutic hypothermia unless bystanders witnessed the cardiac arrest and initiated an adequate basic life support immediately. Also, MI patients with contraindications against hypothermia (e.g., highly elevated bleeding risk) and patients who showed early signs of awakening shortly after return of spontaneous circulation (ROSC) were included into the control group. In all other patients after OHCA, therapeutic hypothermia was induced by cold saline infusion and sustained via intravasal catheter cooling. Because of this selection bias, the study is not designed to compare clinical outcomes between hypothermic and non-hypothermic patients. After reaching the target temperature of 33 ± 1 °C, hypothermia was continued for 24 h followed by controlled rewarming at max. 0.5 °C/h using a standard intravenous cooling catheter (IcyCath^®^, ZOLL, Sunnyvale, CA, USA). Core temperature was measured by a urine thermo-catheter. Sedatives and analgesics (midazolam and sufentanil) were used during hypothermia to suppress shivering.

Survival, neurological outcome in terms of the cerebral performance category scale, and major adverse cardiac events including stent thrombosis were followed at least until hospital discharge. Bleeding events were defined by the BARC bleeding definition. All patients received a close monitoring of important laboratory tests including high-sensitivity troponin T [20]. Patients were followed until discharge of the hospital.

To further elucidate the effects of body temperature on platelet aggregometry, a second cohort of stable patients on dual platelet inhibition was investigated. Only cardio-circulatory stable patients with a regular oral intake of either ticagrelor or clopidogrel were included in this substudy. MI patients received ticagrelor (loading dose 180 mg followed by 90 mg twice a day), whereas patients with a stable coronary disease received clopidogrel (loading dose 300–600 mg followed by 75 mg once a day). Platelet function monitoring was performed by impedance aggregometry >24 h after loading.

### Inclusion and exclusion criteria

Survivors of out-of-hospital cardiac arrest due to a MI (NSTEMI and STEMI) were eligible for the study. Only patients who survived >24 h after admission were included into the study. Exclusion criteria were mainly uncontrolled bleeding or other contraindication for the delivery of ticagrelor. Also, patients with prior intake of a P2Y_12_ antagonist were excluded. Furthermore, oral anticoagulation with a vitamin K antagonist (INR within the therapeutic range) or intake of a direct oral anticoagulant were exclusion criteria. The peri-interventional use of a glycoprotein IIb/IIIa inhibitor (eptifibatide, tirofiban) did not lead to an exclusion of the patient as long as platelet function measurements were more than 8 h apart from the end of the glycoprotein IIb/IIIa inhibitor therapy [21].

### Platelet impedance aggregometry

Platelet function monitoring was performed by impedance aggregometry 24 h after admission, when all patients had received at least two doses of ticagrelor. All patients in the hypothermia group were still at 33 °C at the time point of measurements. Electrical aggregometry measures the impedance between a pair of electrodes immersed in diluted whole blood. The increase in impedance (Ω) is associated with the amount of platelet aggregates deposited on the electrodes after the addition of a platelet agonist [22]. Details of the aggregometry method have been reported before [23, 24]. Aggregation was analyzed by the use of a CA560-CA lumi-aggregometer from Chrono-Log. Aggregometry was performed at 37 °C with a constant stir bar speed of 1000 rpm. In brief, a 0.5 mL aliquot of citrate-anticoagulated whole blood was diluted with 1 volume of prewarmed (37 °C) NaCl (9 g/L) in a polycarbonate cuvette. The electrodes were then immersed in the diluted blood sample and incubated at 37 °C for at least 2 min. After electrical calibration, aggregation was started by the addition of ADP to obtain a final concentration of 5 or 20 µmol/L. The increase of impedance (Ω) was recorded for 7 min. Only 6-min impedance values were used for the analyses. ADP (1 mmol/L; Chrono-Par^®^ reagent no. 070212), cuvettes, and siliconized stir bars were purchased from Probe&Go GmbH (Osburg, Germany). An increase in impedance of <6 Ω was considered as good responsiveness to ticagrelor. An increase of ≥6 Ω was classified as insufficient platelet inhibition with ticagrelor.

### Statistical analysis

Data are presented as mean ± standard deviation (SD). A *p* value <0.05 was considered statistically significant (IBM^®^ SPSS^®^ Statistics, Version 21.0.0). A one-sided binomial test (G*Power 3.1, Institute for Experimental Physics, University of Düsseldorf, Germany) was used to determine a sample size of *n* = 23 hypothermic patients for a given power (1 − *β*) = 0.9 and *α* = 0.05, assuming that enteral delivery of ticagrelor results in insufficient platelet inhibition in less or equal to 30 % of hypothermic patients (effect size = 0.3). For post hoc validation, we calculated a two-sided binomial test (*r* statistics 3.0.2). A paired *t* test was used to compare differences in impedance between hypothermic and normothermic samples (IBM^®^ SPSS^®^ Statistics, Version 21.0.0).

## Results

### General and cardiac outcomes

A total of consecutive 38 patients with STEMI or NSTEMI after OHCA were included into the analysis (30 male and 8 female patients; ages 42 to 91 years; Table 1). Of these primary OHCA survivors, about a third died despite maximum intensive care treatment (intrahospital mortality 36.8 %). 24 patients could be discharged from hospital. Using the Utstein reporting guidelines for the cerebral performance category (CPC) for neurologic outcome [25], 17 patients (44.7 %) were classified as CPC 1 or CPC 2.

**Table 1:** Patient demographics

	Hypothermia (*n* = 27)	No hypothermia (*n* = 11)
Age (years)	61.4 ± 10.5	64.6 ± 10.9
STEMI vs. NSTEMI (*n*)	11 (40.7 %) vs. 16 (59.3 %)	5 (45.5 %) vs. 6 (54.4 %)
LVF		
Normal to mildly impaired (*n*)	3 (11.1 %)	2 (18.2 %)
Moderately impaired (*n*)	9 (33.3 %)	3 (27.3 %)
Highly impaired (*n*)	15 (55.5 %)	6 (54.5 %)
3 vessel CAD (*n*)	18 (66.7 %)	7 (63.6 %)
Time to ROSC (min)	24.7 ± 12.4	18.6 ± 12.2
Discharge from hospital (*n*)	16 (59.3 %)	8 (72.7 %)
Neurologic outcome		
CPC 1 and CPC 2 (n)	10 (37.0 %)	7 (63.6 %)
CPC 3 and CPC 4 (n)	6 (22.2 %)	1 (9.1 %)
Gastroesophageal reflux (mL)	79.6 ± 127.3	203.0 ± 314.8
Lysis or GPIIa/IIIb inhibitor treatment		
Tenecteplase (*n*; time to measurement)	3 (11.1 %); 30.8 ± 3.9 h	2 (18.2 %); 31 ± 1.1 h
Eptifibatide (*n*; time to measurement)	2 (7.4 %); 30.5 ± 2.1 h	0 (0 %); n/a
Tirofiban (*n*; time to measurement)	3 (11.1 %); 21.7 ± 1.5 h	1 (9.1 %); 34 h
Dialysis	0	1 (9.1 %)
Temp. on admission (°C)	35.0 ± 1.0	35.6 ± 1.4
Temp. at loading with ticagrelor (°C)	34.5 ± 1.1	36.0 ± 1.4

*CAD* coronary artery disease, *CPC* cerebral performance category, *Dialysis* dialysis for acute or chronic kidney failure, *LVF* left ventricular function, *ROSC* return of spontaneous circulation, *STEMI* ST-elevation myocardial infraction, *Temp.* temperature

There were no cases of stent thrombosis, recurrent MI, or unscheduled re-angiography within the hospital stay. Most deaths were attributed to fatal hypoxic brain damage, while other patients died despite maximum intensive care treatment in a catecholamine refractory cardiogenic shock. Of note, in none of the 27 patients, hypothermia had to be discontinued ahead of schedule.

On admission, all but three patients had elevated white blood cell counts. C-reactive protein levels were within the normal range or mildly elevated in most patients on admission, but started to increase within 24 h (Table 2).

**Table 2:** Serum chemistry, blood count and blood gas analysis on admission and at the time point of platelet function measurements

	Normal range	On admission		At the time point of measurements	
		Hypothermia (*n* = 27)	No hypothermia (*n* = 11)	Hypothermia (*n* = 27)	No hypothermia (*n* = 11)
CRP (mg/L)	<5 mg/L	9.23 ± 15.24	19.53 ± 15.6	98.64 ± 79.96	101.96 ± 16.61
WBC (nL^−1^)	4 to 10/nL	17.30 ± 6.91	18.94 ± 6.92	12.54 ± 5.65	16.77 ± 6.92
RBC (g/dL)	12 to 15 g/dL	14.07 ± 1.90	12.50 ± 2.65	12.55 ± 2.02	11.21 ± 1.18
TC (nL^−1^)	150 to 440/nL	249.0 ± 55.2	318.3 ± 130.4	212.0 ± 60.0	334.6 ± 159.5
pH	7.37 to 7.45	7.21 ± 0.17	7.18 ± 0.11	7.34 ± 0.10	7.40 ± 0.08
BE (mmol/L)	−2 to +3 mmol/L	−9.46 ± 6.35	−6.16 ± 4.58	−10.33 ± 5.51	−3.45 ± 3.33
Lactate (mg/dL)	<16 mg/dL	52.91 ± 26.79	85.07 ± 49.49	29.53 ± 29.03	22.26 ± 17.69
Hs-TnT (pg/mL)	<50 pg/dL	876.1 ± 1348.2	316.5 ± 371.6	3520.4 ± 8503.9^a^	2898.9 ± 4063.8^a^

Data presented as mean ± SD

*BE* base excess, *CRP* c-reactive protein, *RBC* red blood count, *TC* thrombocytes, *hs-TnT* high-sensitivity troponin T, *WBC* white blood count

^a^ *Hs-*TnT measured at day 3 after admission

### Platelet aggregation

**Fig. 1:**
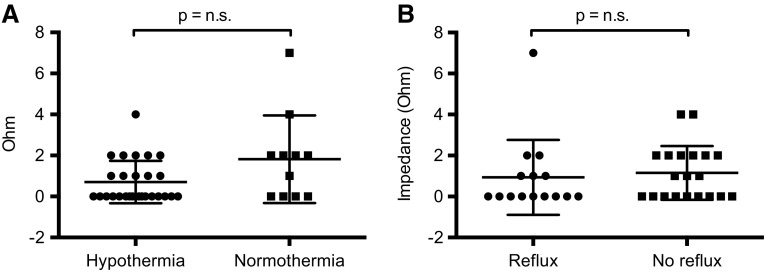
Efficacy of crushed ticagrelor in MI patients after OHCA in pre-specified subgroups. **a** Results of the impedance aggregometry 24 h after admission in *n* = 27 hypothermic patients at 33.0 °C body temperature and *n* = 11 normothermic patients. **b** Results of the impedance aggregometry 24 h after admission in *n* = 15 patients with >50 mL gastroesophageal reflux within the first 6 h after admission and *n* = 20 patients with <50 mL reflux

**Fig. 2:**
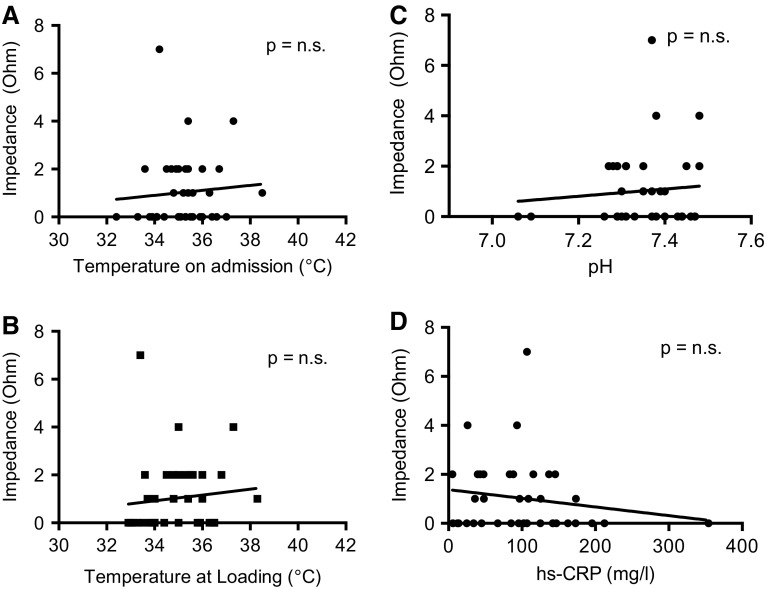
Effects of body temperature, acidosis and inflammation on platelet inhibition by ticagrelor. Correlation between the impedance measured by platelet aggregometry and the body temperature **a** on admission, **b** at the time point of loading with ticagrelor, **c** pH and **d** hs-CRP, respectively

Platelet function was measured by impedance aggregometry 25.6 ± 13.6 h after OHCA. 37 out of 38 (97.4 %) patients had a sufficient platelet inhibition within 24 h after admission. In the hypothermia group, impedance aggregometry showed a good efficacy of ticagrelor in all patients (Fig. 1a). In the non-hypothermic group, one patient with significant gastroesophageal reflux had insufficient platelet inhibition by ticagrelor 24 h after admission. Platelet function was measured after re-application of a loading dose of ticagrelor (180 mg) and showed sufficient inhibition in this patient at 48 h. Other than that there were no hints that gastroesophageal reflux significantly affects platelet inhibition by ticagrelor (Fig. 1b). There was no significant correlation between the impedance measured by platelet aggregometry and neither the core body temperature on admission nor the body temperature at the time point of loading with ticagrelor (Fig. 2a + b). Furthermore, there neither was an association between impedance and hs-CRP as a marker for inflammation nor between impedance and pH as a surrogate parameter for acidosis (Fig. 2c + d).

**Fig. 3:**
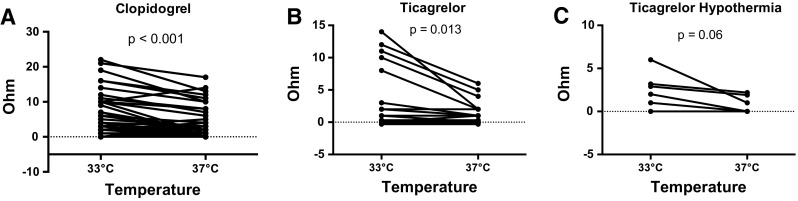
Influence of the temperature of the instrument and the blood sample on impedance. Results of the impedance aggregometry in **a** *n* = 65 normothermic, stable patients receiving clopidogrel; **b** *n* = 20 normothermic, stable MI patients receiving ticagrelor; and **c** *n* = 6 hypothermic patients after OHCA receiving ticagrelor

To assess how the temperature of the instruments and blood samples affect the aggregometry results, we compared the platelet aggregation at 33 and at 37 °C in a separate cohort of cardio-circulatory stable patients on dual platelet inhibition (Fig. 3a + b). There was a strong correlation between the paired samples at 33 and 37 °C for clopidogrel (*n* = 66; *R* = 0.875; *p* < 0.001) and ticagrelor (*n* = 19; *R* = 0.847; *p* < 0.001), respectively. The mean impedance was significantly higher in the cooled samples than in the samples at body temperature for clopidogrel (4.61 ± 5.51 vs. 2.68 ± 4.11 Ω; *p* < 0.001) and for ticagrelor (3.52 ± 4.81 vs. 1.37 ± 1.81 Ω; *p* = 0.013). Eight normothermic patients (12.1 %) receiving clopidogrel had sufficient platelet inhibition at 37 °C, while cooling of the sample to 33 °C suggested insufficient platelet inhibition. With regard to ticagrelor, three normothermic patients (15.8 %) would have been reclassified as poor responders after cooling the blood samples. A shift toward higher impedance at lower body temperature was also observed in hypothermic patients, although the difference did not reach statistical significance (Fig. 2c).

### Bleeding events

A total of four patients (17.4 %) experienced bleeding complications after admission. One patient in the hypothermia group suffered an upper gastrointestinal bleeding with a drop in hemoglobin of >5 g/dL, counting as a type 3a bleeding according to the definition of the Bleeding Academic Research Consortium (BARC) [26, 27]. One patient in the non-hypothermia group started to bleed at the puncture site after coronary angiography following prior out-of-hospital systemic thrombolysis and had a drop in hemoglobin of >3 g/dL (BARC type 2 bleeding). Two patients (one in each group, respectively) experienced less severe complications at the groin (one aneurysm at the puncture site and the other a large hematoma; BARC type 2 bleedings). Two out of four patients (50 %) with significant bleeding complications died during hospital care, but none of the deaths was attributed to bleeding.

## Discussion

In this study, we showed that enteral delivery of crushed and suspended ticagrelor via a nasogastric tube in MI patients after OHCA is effective in vitro and in vivo. Induction of therapeutic hypothermia influenced the results of impedance aggregometry. However, this did not lead to a major loss of efficacy. Likewise, gastroesophageal reflux, acidosis and elevated inflammatory markers did not result in a significant decrease in aggregation efficacy of ticagrelor in most post-cardiac arrest patients. However, one patient with pronounced reflux in the non-hypothermia group showed inadequate platelet inhibition at 24 h. Therefore, especially in patients with a large amount of reflux and enteral delivery of a P2Y_12_ antagonist, platelet function measurements should be considered.

To our knowledge this is the largest cohort of MI patients after OHCA on dual platelet inhibition with ticagrelor. As an additional strength, the impact of temperature on platelet aggregometry was evaluated. Furthermore, to have comparable pathophysiological conditions in the hypothermia group and the control group, only MI patients after OHCA, when injury pathways are still active, were included into the study [4]. In contrast, the control group in two previous studies investigating platelet inhibition in hypothermia included either stable MI patients without a history of cardiopulmonary resuscitation [28] or consisted of patients without MI and without dual platelet inhibition [29].

Overall survival after OHCA remains low despite advances in medical therapy and device technology [30, 31]. Of primary survivors admitted to hospital still more than half of the patients die during their hospital stay [30, 32, 33]. There were no recurrent atherothrombotic events like stent thrombosis, re-infarctions or unscheduled re-angiographies in none of the groups indicating a good clinical efficacy of the dual antiplatelet therapy. Overall, there were no early outcomes that pointed toward a clinical drug resistance to ticagrelor in hypothermic patients after OHCA. This study cannot provide long-term clinical endpoints. However, after termination of artificial respiration, resumption of oral drug intake, and normalization of inflammation and acidosis, a similar long-term efficacy as in previous trials can be assumed [2].

We observed an inversely proportional relationship between the blood temperature and the level of impedance measured by platelet aggregometry. Blood samples taken from hypothermic patients exhibited a higher impedance at their actual temperature of 33 °C than after rewarming to a normal human body temperature of 37 °C. Likewise, impedance in blood samples of normothermic patients taking ticagrelor increased after cooling the samples to 33 °C. The observed increase in impedance in hypothermic patients may reflect a partial loss of efficacy of ticagrelor at lower temperatures. In previous trials, this observation was mostly explained by a decreased bioavailability of ticagrelor due to an impaired GI tract with reflux, decreased GI motility and absorption dysfunction [28, 29]. However, the profound platelet inhibition in rewarmed blood samples of hypothermic patients suggests a sufficient bioavailability of ticagrelor also after enteral delivery in the post-cardiac arrest phase. We observed a shift in impedance in the same direction after cooling blood samples of stable, normothermic patients. Thus, ticagrelor as a reversible allosteric regulator of the P2Y_12_ receptor might undergo changes in its binding characteristics that negatively affect the drug–receptor interaction in hypothermia and in cooled blood of normothermic patients. On the other hand, we observed similar effects on clopidogrel, which is an irreversible inhibitor of the P2Y_12_ receptor. Therefore, pharmacokinetic alterations in the drug–receptor interaction cannot fully explain the increase in impedance and potential loss of efficacy after cooling the blood samples of these normothermic patients with a regular oral clopidogrel intake.

It is a major issue of all tests used to assess platelet function that there is no validation of the different testing systems in hypothermic patients [34, 35]. Several patients on ticagrelor treatment would have been reclassified as poor responders after cooling their blood. However, it is unknown if the seemingly lower degree of platelet inhibition in cooled blood translates into a worse clinical outcome. As we did not observe recurrent atherothrombotic events, we hypothesize that there might be a shift in standard values at lower temperatures for impedance aggregometry.

A recent study by Joffre revealed a high incidence of stent thrombosis (10.9 %) in patients with induced mild hypothermia after OHCA, regardless of the type of P2Y_12_ antagonists [36]. However, only half of the patients were pretreated with acetylsalicylic acid and heparin and none of the patients was loaded with a P2Y_12_ antagonist before PCI and initiation of hypothermia. Similar results have been reported in post-cardiac arrest patients receiving clopidogrel [37]. The question was even raised whether brain salvage using induced hypothermia should be given priority to the prevention of stent thrombosis [28, 37]. Importantly, most deaths in this study were attributed to severe hypoxic brain damage. Therefore, therapeutic strategies for brain salvage are essential for patient outcome. Of note, the mean time from primary PCI to thrombotic event in a study of Penela et al. was 174 h, while hypothermia is usually maintained for 24 (−48 h) only [37]—indicating that most thrombotic complications occur long after rewarming. This may question the hypothesis that acute and temporary changes in blood coagulation and platelet function during the early post-cardiac arrest phase are the main reasons for the incidence of stent thrombosis. In contrast, we and others observed a much lower incidence of stent thrombosis, even if hypothermia had been started before PCI [38, 39]. These differences might be due to variations in out-of-hospital medical treatment and other locally driven factors. In this study, all patients without exception received an out-of-hospital pre-treatment with a full dose of heparin and ASA. In addition, patients with a suboptimal coronary flow after revascularization and a high thrombotic burden received a short-time treatment with a GPIIa/IIIb inhibitor. Unless there were bleeding complications, patients also received low-dose heparin along with their dual antiplatelet therapy during their stay on the intensive care unit.

Furthermore, the time point and the body temperature at loading with a P2Y_12_ antagonist might influence the incidence of stent thrombosis as well. In the ATLANTIC trial, the rate of stent thrombosis in the group that received a prehospital loading with ticagrelor was lower than in the in-hospital group [40]. However, until there will be sufficient data regarding intravenous P2Y_12_ antagonists, a prehospital loading will not be applicable. On admission, most patients were already in a mild hypothermia. Even more important, there was no correlation between the degree of platelet inhibition and the body temperature at the time point of loading with ticagrelor. Starting or rather maintaining hypothermia is possible before loading with ticagrelor. Therefore, platelet inhibition appears to be no reason to hastily abandon neuroprotective hypothermia or to postpone its onset. Nevertheless, further pharmacokinetic analyses and clinical trials are necessary to assess if the results of the in vitro tests de facto reflect a partial loss of efficacy in cooled blood or if the threshold to separate between responders and non-responders might need to be redefined in hypothermic patients.

There are several limitations of this study. Impedance aggregometry is a well-established method for testing platelet function and response to platelet inhibitors. However, it would have been of advantage to confirm our results with other functional platelet tests. Also, we conducted no serial platelet function tests to assess the minimum time interval between loading with ticagrelor and achievement of sufficient platelet inhibition.

In conclusion, we demonstrated that enteral delivery of ticagrelor is effective in vitro and in vivo in MI patients after OHCA. Based on our data, platelet inhibition is no reason to withhold induced hypothermia in patients during the early ROSC phase.
